# Supplementary material for: Understanding Why All Types of Motivation Are Necessary in Advanced Anaesthesiology Training Levels and How They Influence Job Satisfaction: Translation of the Self-Determination Theory to Healthcare
Source: Healthcare (Basel). 2021 Mar 1;9(3):262. doi: 10.3390/healthcare9030262 (PMC7999734; doi:10.3390/healthcare9030262)
Supplement: Supplementary file 1 [file healthcare-09-00262-s001.pdf]

Supplement

Structure of the anaesthesiology department of the University Medical Center Hamburg-Eppendorf, Germany

In Germany, anaesthesia residents are deployed as complete personnel under supervision. In our department, all operating theatres are clustered in four operating rooms (OR) *(see Supplement Figure 1)*. Four anaesthesiologists of different training status or attendings are aligned to the four OR of each cluster. They are supervised by one consultant and one supervising attending.

Anaesthesia induction of following patients is often performed parallel to the proceeding surgery to keep turnover times as short as possible.

Residents are exchanged by the consultant/ supervising attending for breaks and for performing following patients` anaesthesia induction. This is not always possible, as junior residents must be directly supervised during anaesthesia induction. Each medical decision has to be discussed with the consultant/ supervising attending.

The night-shift team consists of one consultant (on call duty) and one in-site supervising attending, one 3rd year and one 4th or 5th year residents. Two further anaesthesiologists, in any year of residency are on call. Anaesthesiological procedures for cardiac surgery are covered by a resident or attending and another on call duty consultant.

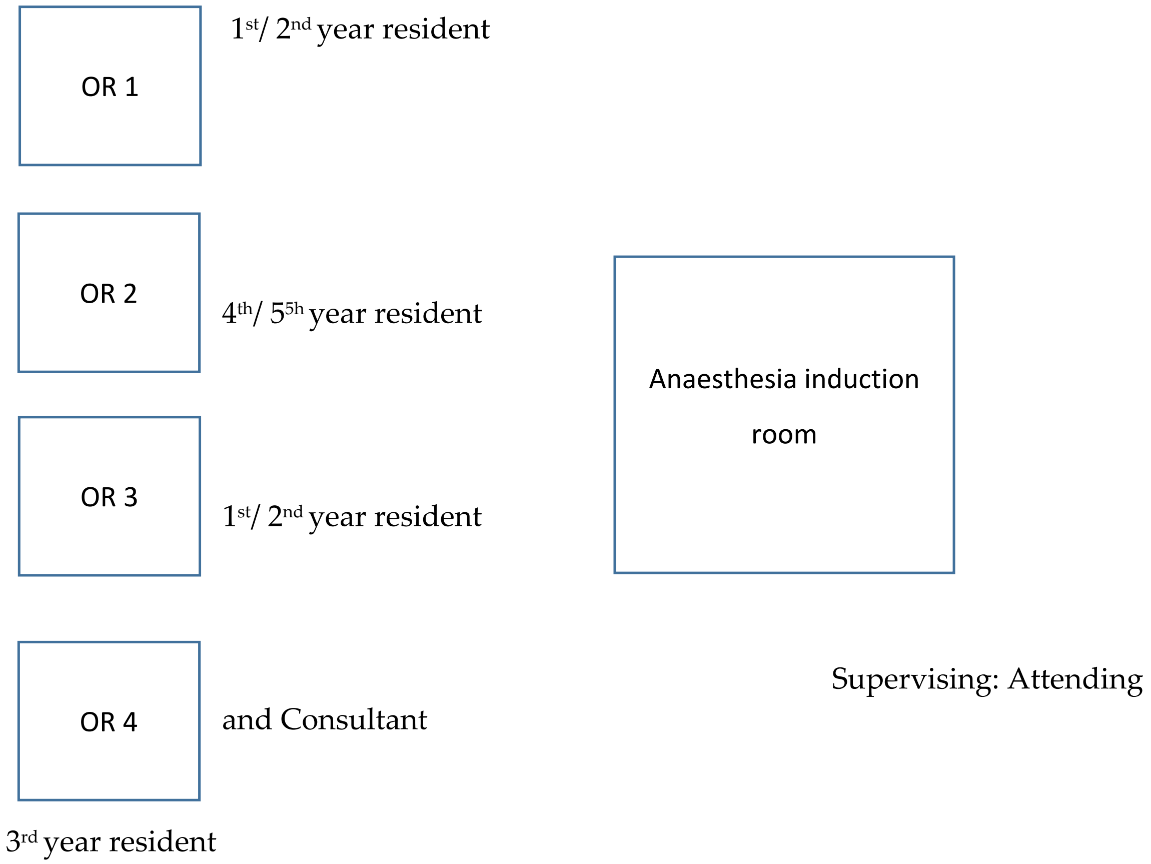

Supplement Figure 1: Schematic example of an OR cluster and its aligned residents

Fragebogen (SIMS)

Lesen Sie jede Aussage aufmerksam durch. Kreuzen Sie jeweils die Nummer an, die am besten beschreibt, warum Sie gerade hier beschäftigt sind.

Warum führen Sie gerade diese Tätigkeit (Anästhesiologie) aus?

	Trifft überhaupt nicht zu	Trifft fast nicht zu	Trifft ein wenig zu	Trifft teilweise zu	Trifft eher zu	Trifft weitgehend zu	Trifft vollkommen zu
1. Weil ich denke, dass diese Tätigkeit interessant ist.	①	②	③	④	⑤	⑥	⑦
2. Weil ich es für mich selbst mache.	①	②	③	④	⑤	⑥	⑦
3. Weil ich es tun soll.	①	②	③	④	⑤	⑥	⑦
4. Es mag gute Gründe geben das hier zu tun, aber ich persönlich sehe keine.	①	②	③	④	⑤	⑥	⑦
5. Weil ich mich schlecht fühlen würde, wenn ich es nicht täte.	①	②	③	④	⑤	⑥	⑦
6. Weil ich denke, dass diese Tätigkeit angenehm ist.	①	②	③	④	⑤	⑥	⑦
7. Weil ich denke, dass diese Tätigkeit gut für mich ist.	①	②	③	④	⑤	⑥	⑦
8. Weil es etwas ist, das ich tun muss.	①	②	③	④	⑤	⑥	⑦
9. Ich tue es, aber ich bin nicht sicher, ob es das wert ist.	①	②	③	④	⑤	⑥	⑦
10. Weil ich bei dieser Sache erfolgreich sein möchte, sonst würde ich mich sehr schämen.	①	②	③	④	⑤	⑥	⑦
11. Weil diese Tätigkeit Spaß macht.	①	②	③	④	⑤	⑥	⑦
12. Weil ich das so entschieden habe.	①	②	③	④	⑤	⑥	⑦
13. Weil ich keine andere Wahl habe.	①	②	③	④	⑤	⑥	⑦
14. Ich weiß nicht; ich sehe nicht, was mir diese Tätigkeit bringt.	①	②	③	④	⑤	⑥	⑦
15. Weil ich im Leben ein “Gewinner” sein möchte.	①	②	③	④	⑤	⑥	⑦
16. Weil ich mich bei dieser Tätigkeit gut fühle.	①	②	③	④	⑤	⑥	⑦
17. Weil ich glaube, dass diese Tätigkeit wichtig für mich ist.	①	②	③	④	⑤	⑥	⑦
18. Weil ich fühle, dass ich es tun muss.	①	②	③	④	⑤	⑥	⑦

19. Ich übe diese Tätigkeit aus, bin aber nicht sicher, ob es gut ist, ihr nachzugehen.	①	②	③	④	⑤	⑥	⑦
20. Weil ich bei dieser Sache sehr gut sein möchte, sonst wäre ich sehr enttäuscht.	①	②	③	④	⑤	⑥	⑦

Supplement figure 2. German SIMS questionnaire, translated by Knörzer and collegues

Note: The motivational qualities (subscale) are computed based on the corresponding items (four items per subscale). Intrinsic: 1, 6, 11, 16; identified: 2, 7, 12, 17; extrinsic: 3, 8, 13, 18; introjected: 5, 10, 15, 20; amotivation: 4, 9, 19, 14
